# Targeting ferroptosis, a novel programmed cell death, for the potential of alcohol-related liver disease therapy

**DOI:** 10.3389/fphar.2023.1194343

**Published:** 2023-05-05

**Authors:** Jing-Fen Shi, Yu’e Liu, Yan Wang, Ru Gao, Yi Wang, Jun Liu

**Affiliations:** ^1^ Institute for Health Policy and Hospital Management, Sichuan Academy of Medical Science and Sichuan Provincial People’s Hospital, University of Electronic Science and Technology of China, Chengdu, China; ^2^ Wenjiang District People’s Hospital of Chengdu, Chengdu, China; ^3^ Tongji University Cancer Center, Shanghai Tenth People’s Hospital of Tongji University, School of Medicine, Tongji University, Shanghai, China; ^4^ Department of Critical Care Medicine, Sichuan Academy of Medical Science and Sichuan Provincial People’s Hospital, University of Electronic Science and Technology of China, Chengdu, China; ^5^ Department of Ultrasound Medicine, Sichuan Academy of Medical Science and Sichuan Provincial People’s Hospital, University of Electronic Science and Technology of China, Chengdu, China

**Keywords:** ferroptosis, ALD, ROS, GPx4, p53, ferroptosis inducers, ferroptosis inhibitors

## Abstract

Ferroptosis is a new iron-dependent cell death mode, which is different from the other types of programmed cell death, such as apoptosis, necrosis, and autophagy. Ferroptosis is characterized by a process in which fatal lipids from lipid peroxidation accumulate in cells and eventually lead to cell death. Alcohol-related liver disease (ALD) is a type of liver injury caused by excessive alcohol intake. Alcohol-related liver disease is a broad-spectrum disease category, which includes fatty liver, steatohepatitis, hepatitis, cirrhosis, and hepatocellular tumors. Recent studies have found that ferroptosis is involved in the pathological development of non-viral liver diseases. Therefore, ferroptosis may be an ideal target for the treatment of non-viral liver diseases. In this review article, we will elaborate the molecular mechanism and regulatory mechanism of ferroptosis, explore the key role of ferroptosis in the Alcohol-related liver disease process, and summarize the existing targeted ferroptosis drugs and their feasibility for the treatment of Alcohol-related liver disease.

## 1 Introduction

Alcohol-related liver disease (ALD) refers to liver damage due to excessive alcohol consumption. It involves a broad spectrum of diseases that includes liver steatosis, steatohepatitis, hepatitis, cirrhosis, and hepatocellular carcinoma (HCC) (Liu et al., 2021a). More than 75 million people have been diagnosed with an alcohol use disorder and are at risk for alcohol-related liver disease (Asrani et al., 2019). Recent studies revealed that ferroptosis plays a number of important roles in alcoholic liver disease and ferroptosis is an ideal target for non-viral liver disease (Liu et al., 2020; Jia et al., 2021; Li et al., 2022; Kouroumalis et al., 2023). As a new mode of programmed cell death, ferroptosis is mainly characterized by the accumulation of intracellular lipid reactive oxygen species (ROS) as well as lipid peroxidation. Cells undergoing ferroptosis typically exhibit abnormal mitochondrial morphology, including mitochondrial shrinkage, increased membrane density, and disruption of the outer mitochondrial membrane. Importantly, iron overload has been reported to be correlated with chronic liver diseases, especially ALD (Ali et al., 2022; Kouroumalis et al., 2023). Therefore, in this review, we will address the canonical signaling pathway of ferroptosis as well as its correlation with the pathogenesis of ALD.

## 2 The pathology of alcohol-related liver disease

Alcohol enters the blood circulation through the gastrointestinal tract, reaches the liver, and is ultimately metabolized by hepatocytes (Teschke, 2018). In hepatocytes, there are three main mechanisms for metabolizing alcohol (Buchanan and Sinclair, 2021), and the first and most important mechanism is ethanol oxidation by alcohol dehydrogenase (ADH) to produce toxic acetaldehyde (Liu et al., 2020). The second route is the micro ethanol oxidation system (MEOS). This system mainly oxidizes ethanol to acetaldehyde and produces ROS through the cytochrome P450 2E1 enzyme (CYP2E1), which triggers oxidative stress and inflammation (Pinero et al., 2018; Teschke, 2018; Zha et al., 2021). The third pathway is the oxidation of ethanol to acetaldehyde by heme-containing catalase (Buchanan and Sinclair, 2021).

The potential molecular mechanisms for ALD development mainly include: direct toxicity and lipid peroxidation of ethanol to hepatocytes, oxidative stress and ROS production, activation of the immune response and accumulation of cytokines, and liver metabolic disorder (Meroni et al., 2018; Kong et al., 2019; Jiang et al., 2020).

Long-term drinking upregulates cytochrome P450 2E1 (CYP2E1), which leads to an increased acetaldehyde concentration and decreased activity of aldehyde dehydrogenase, thus reducing the oxidation of acetaldehyde, resulting in the accumulation of acetaldehyde, eventually causing direct damage to hepatocytes (Ceni et al., 2014; Meroni et al., 2018). In addition, ethanol and acetaldehyde downregulated adiponectin, signal transducer and activator of transcription 3 (STAT3), and zinc levels, thereby inhibiting AMP-activated protein kinase (AMPK), peroxisome proliferator-activated receptor α (PPARα), and the activity of the target genes of AMPK and PPARα. This eventually led to lipid peroxidation and lipid free radical production, resulting in fatty acid accumulation in the liver (Louvet and Mathurin, 2015; Teschke, 2018; Lee et al., 2020). Recent studies have also found that lipid production through free fatty acids from the liver reduced adipose tissue volume in animal models with long-term alcohol intake (Seitz et al., 2018; Buchanan and Sinclair, 2021).

CYP2E1-mediated or ethanol-induced inflammatory oxidative stress can lead to ROS production, and ROS can bind to different proteins, resulting in corresponding conformational and functional changes (Louvet and Mathurin, 2015; Tan et al., 2020). ROS can also bind directly to DNA to produce a highly carcinogenic extracellular domain ε-DNA adduct. This DNA adduct shows high mutagenic potential for base pairs and gene damage to the body. Therefore, in many studies, ε-DNA adducts were used as markers of DNA damage from lipid peroxidation (Linhart et al., 2014; Seitz et al., 2018). In addition, acetaldehyde mediated the impaired synthesis of glutathione through the downregulation of antioxidant genes, including nuclear factor erythroid 2-related factor 2 (*NRF2*) and thioredoxin, and thus reduced the production of antioxidant factors and detoxification enzymes, which led to a decrease of antioxidant system activity (Louvet and Mathurin, 2015; Mueller et al., 2018).

Long-term alcohol intake can accelerate the transfer of endotoxins, such as lipopolysaccharide, from the intestine to the liver, and stimulate the accumulation of neutrophils and macrophages, which eventually leads to hepatocyte inflammation and systemic damage of liver Kupffer cells (Linhart et al., 2014; Mueller et al., 2018; Tan et al., 2020). In addition, liver injury activates the proliferation of hepatic stellate cells, thus enhancing transforming growth factor-β (TGF-β) secretion and collagen synthesis, resulting in extracellular matrix deposition and late fibrosis (Gao et al., 2017).

**FIGURE 1:**
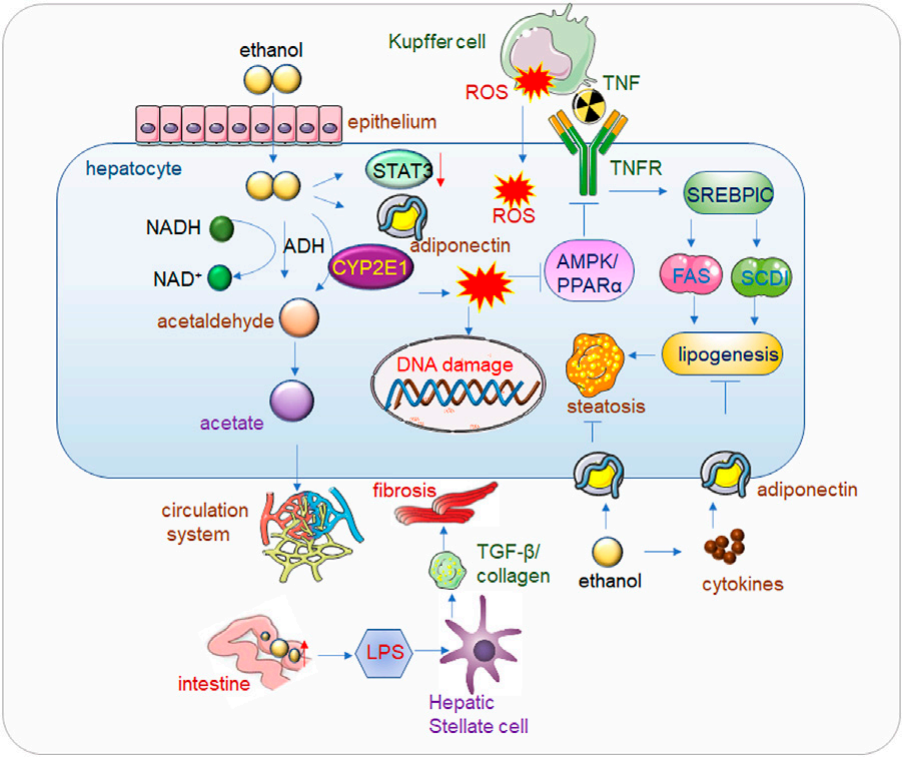
Alcohol related liver disease (ALD). Alcohol passes through the hepatic endothelial cells and enters into the hepatocytes, where the toxic substance acetaldehyde is first generated under the action of alcohol dehydrogenase, and the acetaldehyde is further metabolized into acetate and finally enters the circulatory system to be eliminated. When drinking heavily for a long time, the human body mainly decomposes alcohol through CYP2E1 (Cytochrome P450 2E1) to generate acetaldehyde, and at the same time generates ROS (Reactive Oxygen Species), the accumulation of a large amount of acetaldehyde will reduce the activity of alcohol dehydrogenase, alcohol, acetaldehyde and Accumulation of ROS also reduces the expression of Adiponectin and STAT3, thereby inhibiting AMPK/PPARα, ultimately leading to lipid peroxidation (Lee et al., 2020). In addition, ROS will also cause corresponding damage to DNA. The increase of alcohol in the intestine will increase the permeability of the intestinal lining, so that LPS (Lipopolysaccharide) can enter into liver cells in large quantities and bind to Toll-like receptors (TLRs), mediate the release of cytokines and inflammatory factors, and then increase TGF-β secretion and collagen production, eventually leading to hepatocyte fibrosis. Kupffer cell-derived tumor necrosis factor (TNF) activates sterol regulatory element-binding protein 1c (SERBP1C) by binding to receptors on hepatocytes, thereby increasing lipid production and ultimately leading to fatty liver. Adiponectin can inhibit adipogenesis, but alcohol causes the release of inflammatory factors in adipose tissue, which inhibits Adiponectin, and eventually leads to fatty liver.

A large number of studies have reported that alcohol intake could significantly increase the risk of iron overload and reduced the iron deficiency in liver cells and Kupffer cells (Suzuki et al., 2002; Xiong et al., 2003; Ioannou et al., 2004; Li et al., 2014). Large quantities of free iron and alcohol synergistically induce oxidative stress and lipid peroxidation, thus increasing the expression of transferrin receptor 1 (TfR1), which improves intestinal iron absorption. Therefore, the additive effects of iron absorption and deposition further increase liver injury (Ramm and Ruddell, 2005; Barbier et al., 2019) (Figure 1).

## 3 Mechanism of ferroptosis

Ferroptosis includes three major metabolites: thiols, lipids, and iron ions, and forms an iron-dependent lipid peroxidation that eventually leads to cell death (Parola and Pinzani, 2019). It was initially found that the cystine-import glutathione (GSH)-GPX4 molecular machine played a role in the inhibition of ferroptosis, and the role of pools as the direct executor of ferroptosis was established (Xu et al., 2023). Recent studies have identified a glutathione peroxidase 4 (GPX4)-independent ferroptosis monitoring pathway (Gu et al., 2023). Importantly, these studies have focused on cell metabolism, and the results have shown a close link between ferroptosis and metabolic pathways (Harrison-Findik et al., 2007; Parola and Pinzani, 2019).

### 3.1 Classic GPX4 regulates the ferroptosis pathway

In 2001, the Stockwell laboratory carried out a high-throughput screening experiment of new anti-tumor small-molecule drugs, and found a series of compounds that induced a non-apoptotic, non-necrotic cell death mode (Harrison-Findik, 2007). The results of reverse screening showed that a variety of iron chelating agents and lipid soluble free radical scavenging antioxidants inhibited this form of cell death, which was named ferroptosis because it required iron ions (Silva et al., 2017). Two components of cells were identified in ferroptosis: the human cystine/glutamic acid reversal transporter system Xc^−^, and GPX4 (Jiang et al., 2021; Yan et al., 2021).

In mammalian cells, GPX4 is a major enzyme (Harrison-Findik, 2007; Harrison-Findik et al., 2007) that catalyzes the reduction of phospholipid hydroperoxide (PLOOHs). GPX4 requires its own catalytic selenocysteine and two electrons, generally provided by GSH, to reduce phospholipid and cholesterol hydroperoxide to their corresponding ethanol (Dolma et al., 2003; Yagoda et al., 2007). Conrad’s in-depth and thorough study of the first conditional *Gpx4* knockout mice provided early evidence for the lipid peroxidation-dependent non-apoptotic cell death caused by GPX4.

### 3.2 Peroxidation of phospholipids

Uncontrolled lipid peroxidation is a marker of ferroptosis, and studies in the 1950s pointed to the association between trace elements such as selenium, vitamin E, and cysteine with lipid peroxidation (Dixon et al., 2014; Yang et al., 2014). Lipid peroxidation requires polyunsaturated fatty acid-phospholipids (PUFA-PLs) from the phospholipid bilayer to extract a hydrogen atom between two carbon-carbon double bonds to form a free radical centered on the C atom, and then reacts with an oxygen molecule to produce a hydrogen peroxide radical (Seiler et al., 2008; Maiorino et al., 2018). Genome-wide haploid and clustered regularly interspaced short palindromic repeats (CRISPR)-Cas9 based screening revealed that two membrane remodeling enzymes, acyl-CoA synthetase long chain family member 4 (ACSL4) and lysophosphatidylcholine acyltransferase 3 (LPCAT3), were important drivers of ferroptosis (Schwarz and Foltz, 1999).

The role of ACSL4 in the process of iron death is based on ACSL4 preferentially linking to long-chain PUFAs in the presence of coenzyme A, including arachidonic acid (20:4) and docosahexaenoic acid (22:4), so that they can be re-esterified by many lysophosphatidylcholine acyltransferase (LPCAT) enzymes. The deletion of the *Acsl4* gene resulted in a rapid transition from a long-chain PUFA tail to a short chain and monounsaturated fatty acids, while the same phenomenon was observed in wild-type cells with drug inhibition of ACSL4 activity (Doll et al., 2017). Therefore, inhibition of ACSL4 expression may be the main mechanism of inhibiting ferroptosis. There are still many controversies and uncertainties about how lipid peroxidation occurs (Conrad and Pratt, 2019).

### 3.3 Iron metabolism

The key role played by phospholipid peroxidation in cellular metabolism during ferroptosis explains why it is dependent on iron ions. First, the metabolic enzymes LOXs and POR, which are involved in phospholipid peroxidation, require Fe ions to function as catalysts, and iron ions are also critical for most catalytic enzymes involved in cellular ROS production. Second, non-enzyme-catalyzed and Fe-ion-dependent Fenton chain reactions may be important for ferroptosis: when GPX4 is inhibited, PLOOHs are able to sustain the initiation of Fenton chain reactions for rapid amplification of PLOOHs, which are markers of ferroptosis. These radicals further react with PUFA-PLs to increase the yield of PLOOH (Chen et al., 2021a).

This regulation is mediated mainly through two key proteins in the post-transcriptional network, IRP1 and IRP2, which regulate intracellular iron storage and release, as well as transfer and excretion (Harrison-Findik et al., 2007; Doll et al., 2017). It is conceivable that many intracellular processes change the sensitivity of cells to iron death by changing the active iron content of cells. Recent studies on mouse models *in vivo* further elucidated the important role of iron regulation in ferroptosis. For example, knockout ferritin heavy chain may cause heart disease by enhancing ferroptosis (Fang et al., 2020; Packer, 2023).

### 3.4 GPX4-independent monitoring pathway

By using gene suppression screening or CRISPR-Cas9 synthetic lethal screening, the Conrad and other research groups independently identified an important protein in ferroptosis, ferroptosis suppressor protein 1 (FSP1), which acted from a completely different thiol-dependent axis (Kim et al., 2001; Dixon et al., 2015). Because of the homology between FSP1 and apoptosis inducing factor mitochondria associated 1 (AIFM1), FSP1 was previously named apoptosis inducing factor mitochondria associated 2 (AIFM2) (Kwon et al., 2015; Brown et al., 2019; Fang et al., 2020). Originally considered a pro-apoptotic protein, AIFM1 was found to mediate the proper folding and transport of mitochondrial membrane proteins (Yu et al., 2020). Similarly, FSP1 does not have many apoptotic functions, but protects cells from GPX4 inhibitors and *Gpx4* gene knockout-induced ferroptosis. FSP1 appears in several membrane structures, including the plasma membrane, Golgi, and perinuclear structure. Mutation of the FSP1 will lead to loss of its cell protective function (Doll et al., 2019), and increased expression of FSP1 initiates tumorigenesis even in KRAS-mutated cells at the presence of ferroptosis inducer (Muller et al., 2022). In terms of mechanism, because of its NADH-ubiquinone (CoQ_10_) oxidoreductase activity (Bersuker et al., 2019), FSP1 has inhibits lipid peroxidation and iron death. The mechanism is to produce pantothenol by restoring ubiquinone (CoQ_10_), which in turn directly restores lipid free radicals and ends lipid autoxidation (Lv et al., 2022). Recently published article utilizing NanoString technology revealed that NUPR1 (nuclear protein 1, transcriptional regulator), which is upregulated at the presence erastin (a ferroptosis inducer by depletion of GSH and thereby stimulating GPX4 degradation), is a driver of ferroptosis resistance. By upregulating LCN2 (lipocalin 2) expression, NUPR1 inhibits iron accumulation and subsequent oxidative damage, and ultimately results in the inhibition the ferroptosis mediated cell death (Liu et al., 2021b).

### 3.5 New pathway of p53 dependent cell ferroptosis

**FIGURE 2:**
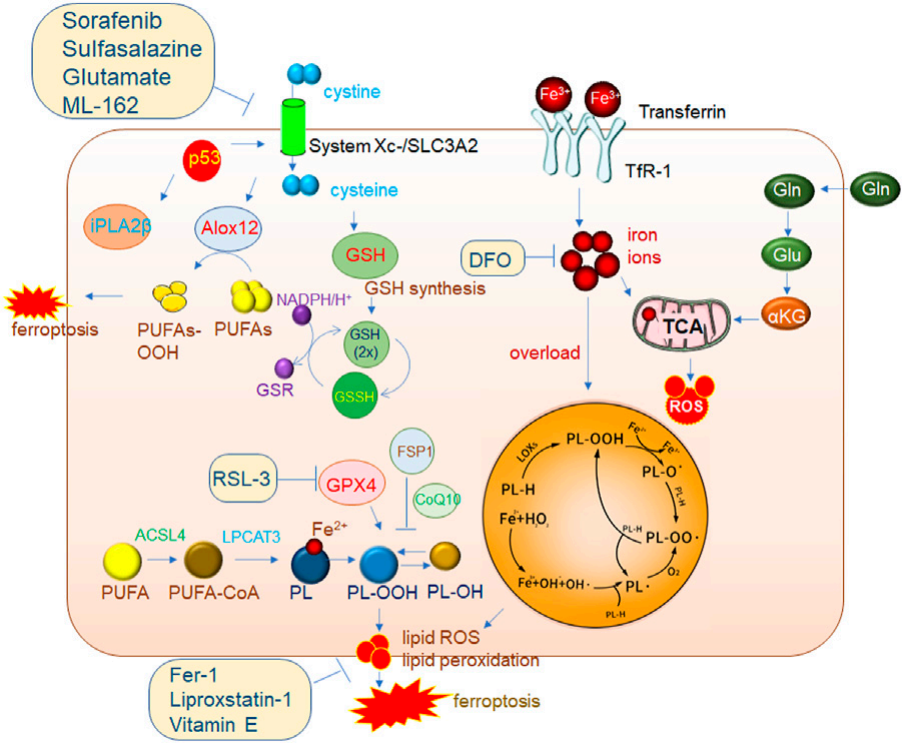
Mechanisms of ferroptosis regulation. The classic GPX4 (glutathione peroxidase 4) pathway. System Xc-brings cystine into cells and is reduced to cystine under the action of GSH, and cystine is an important synthetic raw material for GSH. GPX4 reduces PL-OOH (phospholipid hydroperoxide) to PL-OH in the presence of GSH, thereby inhibiting ferroptosis. ACSL4 (acyl-CoA synthetase long chain family member 4)/LPCAT3 (lysophosphatidylcholine acyltransferase 3) is a very important driver of ferroptosis. PUFA (polyunsaturated fatty acid) is catalyzed by ACSL4 to generate PUFA-CoA, which is then catalyzed by LPCAT3 to generate PL (phospholipid), which is finally catalyzed by LOXs (lipoxygenase) to generate PL-OOH, and then lipid peroxidation occurs, eventually leading to The membrane ruptures, driving iron death. 2. The accumulation of a large amount of iron ions in cells can also induce ferroptosis. 3. P53-mediated ferroptosis pathway. One of the pathways is to inhibit the expression of SLC7A11 (solute carrier family 7 member 11) in the System Xc-complex, thereby inhibiting GSH synthesis, resulting in the inhibition of GPX4 activity and ultimately the induction of ferroptosis. In addition, decreased expression of SLC7A11 increases free ALox12 (lipoxygenase12), which catalyzes the production of PUFAs-OOH from PUFAs. Another pathway is to inhibit the activity of ALox12 through the downstream target gene iPLA2β of p53, thereby inhibiting the occurrence of ferroptosis. This also shows that P53 plays an important role in the process of ferroptosis.

Since its discovery in 1979, p53 has been one of the focuses of oncology. In fact, p53 also plays an important role in tumors and the recently discovered induction of cell ferroptosis (Wu et al., 2002). The Gu Wei laboratory published an article in 2015, which first revealed that p53 inhibited tumor development by promoting cell ferroptosis (Ohiro et al., 2002). Cystine is a dimer of cysteine. Cysteine is an important component in the synthesis of GSH, and GSH is the main donor restoring power to the ferroptosis inhibitor protein GPX4. The deficiency of Cys leads to a deficiency of GSH synthesis, which affects the normal function of GPX4 and leads to cell ferroptosis. P53 was shown to inhibit solute carrier family 7 member 11 (SLC7A11) expression at transcriptional level, thus promoting cell ferroptosis and leading to tumor inhibition. Further studies showed that the acetylation of the p53 K101 site played an important role in the inhibition of SLC7A11 by p53 (Wang et al., 2021). These results suggest that induction of ferroptosis may be an important weapon for p53 to inhibit tumor growth. A study in 2019 found that when p53 downregulated SLC7A11, lipoxygenase12 (ALOX12) was released. Free ALOX12 oxidizes polyunsaturated fatty acid chains of cell membrane phospholipids, resulting in cell ferroptosis. These results also demonstrate that the p53-SCL7A11 axis promoted ferroptosis in a GSH-independent manner (Susin et al., 1999). Recently, the Gu Wei laboratory study on the *IPL2* gene also showed that it inhibited ferroptosis by cleaving the oxidized cell membrane PUFA. Interestingly, p53 activated iPLA2β when external stimuli were minimal to inhibit ferroptosis (Reinhardt et al., 2020). In particular, it was shown that the p53/SLC7A11/ALOX12 and p53/iPLA2β pathways were independent of GPX4 and ACSL4. These two pathways are different from the classical iron death model proposed by Stockwell and represent a new type of iron death mechanism. In conclusion, the iron death regulated by p53 is a basic iron death pathway which is different from and related to the classical GPX4-centered iron death model (Figure 2).

Although cystatin-GSH-GPX4 is thought to be the primary ferroptosis regulatory system in mammals, recent genome-wide screens have identified ferroptosis mechanisms that do not depend on GPX4 (Yang et al., 2022). By using either a genetic repression screen or a CRISPR-Cas9 synthetic lethality screen, the Conrad and Olzmann groups independently identified an important protein in ferroptosis, FSP1, which functions from a completely different thiol-dependent axis (Magtanong et al., 2019; Zheng and Conrad, 2020; Lange and Olzmann, 2021). A study (2019) identified the lipid oxidase ALOX12 as a key regulator of p53-dependent ferroptosis (Chu et al., 2019). However, SLC7A11 directly binds ALOX12 and thus restricts its function (Koppula et al., 2021). Recently, the Wei Gu laboratory found a new target gene of p53, iPLA2β, which inhibited ferroptosis by cleaving the oxidized cell membrane PUFA (70).

## 4 The correlation between ALD and ferroptosis

In previous review on ALD patients, oxidative stress were found in the liver (Dey and Cederbaum, 2006). Recently, by investigating the hepatic tissues from 31 patients with ALD and 5 normal livers, iron overload in hepatocytes is also a risk factor for liver fibrosis in ALD patients, with the increased TfR expression in hepatocytes of tissues with ALD (Suzuki et al., 2002). In addition, in alcoholic steatohepatitis rat models, iron accumulation was observed in hepatic macrophages/Kupffer cells (KC) with increased transferrin receptor-1 and hemochromatosis transcription and translation, elevated iron uptake and nonheme iron content and decreased response to NF-κB. Moreover, macrophages derived from human PBMC, showed that iron overload stimulate the TNF-α release (Tsukamoto, 2002; Xiong et al., 2008). In severe alcoholic hepatitis (SAH) patients, iron overload in hepatocytes/macrophages triggered a dis-integrin and metalloprotease 17 (ADAM17) activation was investigated by transcriptomics of patient PBMCs. Activated ADAM17 cleaved and released soluble extracellular domains, and then increased tumor necrosis factorα and soluble CD163, thereby activating macrophages, and ultimately enhanced liver inflammation. Therefore, serum iron, sCD163 and inflammatory cytokine as TNF-α correlate with outcome of SAH patients (Maras et al., 2018). In general, previous studies have shown that iron overload is an important factor driving alcohol-induced liver injury (Chu et al., 2019; Gautheron et al., 2020; Chen et al., 2021b). Recently, an *in vitro* study on L-02 cells, a normal human hepatocyte line and *in vivo* study on alcohol induced liver injury C57BL/6 mice, found that alcohol induced hepatocyte death, accompanied by accumulation of lipid peroxidation and decreased expression of SLC7A11 and GPX4 (Xu et al., 2023). *De novo* lipogenesis plays a crucial role in ALD. In the wild type and CYP2E1 KO mice with alcohol induced liver injury, activation of hepatocyte cannabinoid receptor-1 (CB1R) by hepatic stellate cell derived 2-arachidonoylglycerol (2-AG), drives bidirectional signaling between hepatocytes and hepatic stellate cell through SLC7A1/glutamate signaling, and thereafter induce alcoholic steatosis (Choi et al., 2019). Studies on *Lpin1*‐Tg mice with the C57BL/6 background showed that Lipin-1, a magnesium ion-dependent phospholipase, could regulate lipid metabolism through the production of diacylglycerol (DAG) (Brohee et al., 2021). Lipin-1 is closely related to alcoholic steatohepatitis in adipose tissue by overexpression of adipose-specific lipin-1, which in turn accelerates iron accumulation, causes lipid peroxidation, reduces GSH and GAPDH, and promotes ferroptotic liver damage in mice after alcohol administration (Zhou et al., 2019). These results suggest that adipon-1 in adipose tissue induces alcoholic liver injury by driving iron death in hepatocytes. In addition to lipin-1, another mammalian NAD^+^- dependent protein deacetylase, also plays an important role in ALD. In the intestinal-specific *Sirt1* KO mice, increased liver inflammation, iron metabolism and lipid peroxidation disorders were observed, accompanied by normalized liver iron death related genes (such as *Gpx4*, *Acsl4*, and *p53*), thus forming a protective effect on liver injury caused by alcohol (Zhou et al., 2020). In alcohol induced rat liver injury model, inhibition of p62/Nrf2/Keap1/SLC7A11 pathway by fucoidan could protect hepatocytes from ferroptosis and inhibit iron overload (Xue et al., 2022).

Senescence is a cellular process marked with irreversible cell cycle arrest, which limits the proliferative potential of cells. Cellular senescence has been correlated with a varieties of liver diseases, including ALD (Ferreira-Gonzalez et al., 2021). Expression of senescence markers, i.e., hepatocyte p21 expression, has been proved to be negatively correlated with ALD outcomes (Aravinthan et al., 2013). Inhibition of cellular senescence by vitamin C, could alleviate the pathogenesis of ALD (Baek et al., 2022). Yangonin could ameliorate cellular senescence in ALD through the activation of nuclear receptor FXR (Dong et al., 2021). Oroxylin A could inhibit the alcohol induced hepatocyte senescence through the activation of YAP pathway (Jin et al., 2018). These studies suggest that inhibition of cellular senescence could alleviate ALD. Meanwhile, senescence has been reported with iron overload and ferroptosis in liver diseases, especially ALD. Through Fenton reaction, iron overload in the liver could stimulate the liver damage by increasing oxidative stress, activating Kupffer cells (KCs) and hepatic stellate cells (HSCs) and thereby stimulating ferroptosis (Li et al., 2022). Meanwhile, alcohol could stimulate iron absorption and enhance iron uptake in hepatocytes (Rouault, 2003). To a broader definition, alcohol is also detrimental to cells by inducing cellular senescence and aberrant iron metabolism. Studies on bone marrow derived mesenchymal stem cells revealed that alcohol could induce premature senescence by increasing intracellular ROS and upregulate senescent marker genes as p16^INK4a^ and p21^kip1^, and thereby impair osteogenic differentiation (Chen et al., 2017). However, another study revealed that iron accumulation in senescent cells is coupled with the inhibition of ferroptosis. Studies in senescent cells of ageing hepatic tissue revealed that intracellular iron is greatly accumulated with the lysosomal dysfunction and impaired ferritin degradation, and the senescent cells are resistant to ferroptosis (Masaldan et al., 2018). Therefore, targeting cellular senescence by the regulation of iron overload and inducing ferroptosis may be a promising clinical strategy for the ALD treatment.

## 5 Drugs for ferroptosis and the possibility of its use in alcohol-related liver disease

In recent years, increasingly more drugs targeting cell ferroptosis have appeared. Compounds that can precisely regulate ferroptosis play an important role in explaining the mechanism of ferroptosis-related diseases. The first approach is inducers targeting system Xc^−^, including sorafenib (Llovet et al., 2008; Dahlmanns et al., 2017; Ma et al., 2017; Huang et al., 2022), sulfalazine (Tsai et al., 2016; Patel et al., 2019), and glutamate (Bridges et al., 2012; Parker et al., 2021). The second approach is targeting GPX4, such as RAS-selective lethal 3 (RSL3) and ML-162 (Shin et al., 2018; Sui et al., 2018; Li et al., 2021; Liu et al., 2022). The ferroptosis inhibitors, Fer-1 (Miotto et al., 2020; Sripetchwandee et al., 2023), lipoxstatin-1 (Yang et al., 2014; Shin et al., 2018; Fan et al., 2021), and 1,8-Diazafluoren-9-one (DFO) (Bruni et al., 2018; Zeng et al., 2022; Zhou et al., 2022), are the three most frequently used compounds (Table 1).

**TABLE 1:** The Potential target of ferroptosis inducers and inhibitors in alcohol-related liver diseases.

Classifification	Drug	Inducers or inhibitors	Mechanism
Regulating Xc- system	sorafenib	Inducers	Inhibits system Xc-, resulting in GSH depletion
Regulating Xc- system	sulfasalazine	Inducers	Inhibits system Xc-, resulting in GSH depletion
Regulating Xc- system	glutamate	Inducers	gradual accumulation of Glutamate can inhibit the absorption of Cystine by cells through competitive inhibition of system Xc-
GPX4	RSL3	Inducers	Directly inactivates GPX4, resulting in lipid peroxidation
Regulating Xc- system	ML-162	Inducers	gradual accumulation of Glutamate can inhibit the absorption of Cystine by cells through competitive inhibition of system Xc-
PUFAs	Fer-1	Inhibitors	Inhibits lipid peroxidation
Gpx4	liproxstatin-1	Inhibitors	Reduce mitochondrial ROS production, restore
			GPX4 level and inhibit lipid peroxidation
Iron metabolism	DFO	Inhibitors	reduce intracellular iron levels, thereby inhibiting lipid peroxidation
PUFAs	Vitamin E	Inhibitors	inhibits cell ferroptosis by reducing membrane PUFAs oxidation level

### 5.1 Ferroptosis inducers

Sorafenib is a class of kinase inhibitors approved by the food and drug administration (FDA) for the treatment of HCC, and sorafenib has been reported to induce cell ferroptosis in HCC, too (Yang et al., 2016). In addition, sorafenib also inhibits system Xc^−^, which leads to a decrease of GSH and the accumulation of lipid ROS (Dixon et al., 2012). In addition, sorafenib also promotes hepatocyte fibrosis, but whether ferroptosis is involved in hepatocyte fibrosis remains to be further studied (Zilka et al., 2017).

An FDA-approved anti-inflammatory drug for rheumatoid arthritis is sulfasalazine (Llovet et al., 2008; Friedmann Angeli et al., 2014). Sulfasalazine is an antagonist of system Xc^−^, and inhibits the absorption of cystine and thus reduces the synthesis of GSH(89). In addition, sulfasalazine promotes iron death by inhibiting system Xc^−^ in cancer cells (Shin et al., 2018). However, sulfasalazine monotherapy can cause tumor resistance, so it is usually combined with traditional drugs (Chande et al., 2016; Ma et al., 2017).

RSL3 is the first GPX4 inhibitor that causes irreversible inactivation of GPX4 by directly covalently binding to its nucleophilic active site. It was shown that a 2 μM concentration of RSL3 caused cell death (Su et al., 2020). Similarly, compound ML162 is a GPX4 inhibitor that induces ferroptosis by inhibiting GPX4 enzyme activity.

### 5.2 Ferroptosis inhibitors

Ferrostastin-1 (Fer-1) is the first ferroptosis inhibitor, and it can reduce the oxidation of PUFAs in the membrane structure. In addition, many studies focused on the synthesis and activation of PLOOHs, particularly the precursors of PLOOHs, PUFAs, in the ferroptosis state. Non-enzyme-catalyzed, Fe-dependent Fenton chain reactions may be important for ferroptosis: when GPX4 is inhibited, PLOOHs are able to sustain the initiation of Fenton chain reactions in order to rapidly amplify PLOOHs, which are markers of ferroptosis. PLOOHs generate the radicals PLO and PLOO with Fe^2+^ and F^3+^, respectively, which further react with PUFA-PLs to increase the yield of PLOOHs. Because GPX4 is the major PLOOH-neutralizing enzyme, one of the usual molecular mechanisms of erastin and RSL-3-induced ferroptosis is that both compounds inhibit GPX4 activity, except that erastin does so directly, whereas RSL-3 inhibits GPX4 activity by inhibiting cysteine uptake (Shintoku et al., 2017). As a result, this leads to the accumulation of PLOOHs, which in turn cause rapid and irreparable cytoplasmic membrane damage and ultimately cell death (Shintoku et al., 2017). Previous studies showed that Fer-1 ameliorated liver injury by inhibiting ferroptosis in liver cell (Yamada et al., 2020; Jiang et al., 2022).

Vitamin E is also a type of fat-soluble RTA that inhibits cell iron death by reducing membrane PUFA oxidation levels (Yang et al., 2014). Dated back to last century, vitamin E has been proved to alleviate lipid peroxidation for the treatment of alcoholic liver injury in murine models (Nanji et al., 1996). Further murine studies on vitamin E revealed that it could mitigate the toxic effects of alcohol by reducing oxidative stress (Kaur et al., 2010). In 2016, Carlson et al. found that vitamin E enabled hepatocyte-specific *Gpx4*-silenced mice to survive normally (Dixon et al., 2014). These results suggest that vitamin E may improve hepatocyte degeneration by inhibiting ferroptosis.

Accumulating evidences revealed the therapeutic potential of ferroptosis inhibitors in chronic liver diseases. In acute-on-chronic liver failure murine models established by carbon tetrachloride, D-galactosamine, lipopolysaccharide or H_2_O_2_, Fer-1 could alleviate lipid peroxidation, repress ferroptosis and play hepatocyte role via the inhibition of Nrf2 (Wu et al., 2022). Ferrostatin-1 could also effectively attenuate liver damage and protected liver structures in autoimmune hepatitis animal models through the regulation of GPX4/Nrf2/HO-1 signaling pathway (Zhu et al., 2021). It play hepatocyte protective role by reducing iron load in thioacetamide-induced acute liver injury murine model (Jiang et al., 2022). DFO could reduce intracellular iron levels and thereby alleviate liver fibrosis induced by CCl4 in rats (Mohammed et al., 2016). It could also dose-dependently reduce the hepatocellular inflammation and improve liver function for more than 500 chronically transfused patients within 1 year of the observation period (Brissot et al., 2005). Liproxstatin-1 could also alleviate fatty liver disease with metabolic dysfunction in mice (Tong et al., 2023). Vitamin E has been well documented for chronic liver diseases as liver fibrosis and nonalcoholic fatty liver disease (Di Sario et al., 2007; El Hadi et al., 2018; Nagashimada and Ota, 2019). However, there still exist controversial on the pharmacological effect of vitamin E on nonalcoholic fatty liver disease (Lavine et al., 2011; Ivancovsky-Wajcman et al., 2019). To date, the majority of the studies on ferroptosis inhibitors are based on animal models, there is a blank for clinical studies on ferroptosis inhibitor in the treatment of both ALD and the other chronic liver diseases.

Although there is a consensus that lipid disorder and iron overload are common features of alcoholic liver disease, it is only in recent years that researchers began to think about the relationship between ferroptosis and alcohol-induced liver injury. In the study of ALD, we found that this type of liver injury is accompanied by cell ferroptosis, and the status of liver injury is significantly improved by inhibiting cell ferroptosis. Previous cell and mouse ALD models showed that alcohol treatment led to the accumulation of ROS and lipid peroxidation, which were compensated by ferroptosis inhibitors. Therefore, we have reason to believe that targeting ferroptosis will be a promising method for the treatment of ALD (Zhou et al., 2019).

## 6 Conclusion and perspectives

Ferroptosis is a cell death mode regulated by iron metabolism and lipid peroxidation pathways. Cell ferroptosis is involved in many diseases, including ALD. The specific role of ferroptosis in ALD is still under study, and as cell ferroptosis itself is a complex process, its potential molecular mechanisms are currently not very clear. Therefore, it is necessary to perform more in-depth research to understand cell ferroptosis and the relationship between cell ferroptosis and disease occurrence.

## References

[B1] AliN.FerraoK.MehtaK. J. (2022). Liver iron loading in alcohol-associated liver disease. Am. J. Pathol. 10.1016/j.ajpath.2022.08.010 36306827

[B2] Aravinthan A. Pietrosi G. Hoare M. Jupp J. Marshall A. Verrill C. (2013). Hepatocyte expression of the senescence marker p21 is linked to fibrosis and an adverse liver-related outcome in alcohol-related liver disease. PLoS One 8 (9), e72904. 10.1371/journal.pone.0072904 24086266 PMC3781134

[B3] Asrani S. K. Devarbhavi H. Eaton J. Kamath P. S. (2019). Burden of liver diseases in the world. J. Hepatol. 70 (1), 151–171. 10.1016/j.jhep.2018.09.014 30266282

[B4] Baek S. M. Lee S. W. Lee Y. J. Choi S. K. Kim H. Y. Seo M. S. (2022). Vitamin C alleviates alcoholic liver injury by suppressing neutrophil infiltration in senescence marker protein 30-knockout mice irrespective of its antioxidant effects. Life Sci. 297, 120228. 10.1016/j.lfs.2021.120228 34921864

[B5] Barbier L. Ferhat M. Salame E. Robin A. Herbelin A. Gombert J. M. (2019). Interleukin-1 family cytokines: Keystones in liver inflammatory diseases. Front. Immunol. 10, 2014. 10.3389/fimmu.2019.02014 31507607 PMC6718562

[B6] Bersuker K. Hendricks J. M. Li Z. Magtanong L. Ford B. Tang P. H. (2019). The CoQ oxidoreductase FSP1 acts parallel to GPX4 to inhibit ferroptosis. Nature 575 (7784), 688–692. 10.1038/s41586-019-1705-2 31634900 PMC6883167

[B7] Bridges R. J. Natale N. R. Patel S. A. (2012). System xc(-) cystine/glutamate antiporter: An update on molecular pharmacology and roles within the CNS. Br. J. Pharmacol. 165 (1), 20–34. 10.1111/j.1476-5381.2011.01480.x 21564084 PMC3252963

[B8] Brissot P. Turlin B. Forni G. L. Alimena G. Quarta G. Selleslag D. (2005). Iron chelation therapy with deferasirox (Exjade®, ICL670) or deferoxamine results in reduced hepatocellular inflammation and improved liver function in patients with transfusion-dependent anemia. Blood 106 (11), 823. 10.1182/blood.v106.11.823.823

[B9] Brohee L. Cremer J. Colige A. Deroanne C. (2021). Lipin-1, a versatile regulator of lipid homeostasis, is a potential target for fighting cancer. Int. J. Mol. Sci. 22 (9), 4419. 10.3390/ijms22094419 33922580 PMC8122924

[B10] Brown C. W. Amante J. J. Chhoy P. Elaimy A. L. Liu H. Zhu L. J. (2019). Prominin2 drives ferroptosis resistance by stimulating iron export. Dev. Cell 51 (5), 575–586. 10.1016/j.devcel.2019.10.007 31735663 PMC8316835

[B11] Bruni A. Pepper A. R. Pawlick R. L. Gala-Lopez B. Gamble A. F. Kin T. (2018). Ferroptosis-inducing agents compromise *in vitro* human islet viability and function. Cell Death Dis. 9 (6), 595. 10.1038/s41419-018-0506-0 29789532 PMC5964226

[B12] Buchanan R. Sinclair J. M. A. (2021). Alcohol use disorder and the liver. Addiction 116 (5), 1270–1278. 10.1111/add.15204 32710592

[B13] Ceni E. Mello T. Galli A. (2014). Pathogenesis of alcoholic liver disease: Role of oxidative metabolism. World J. Gastroenterol. 20 (47), 17756–17772. 10.3748/wjg.v20.i47.17756 25548474 PMC4273126

[B14] Chande N. Townsend C. M. Parker C. E. MacDonald J. K. (2016). Azathioprine or 6-mercaptopurine for induction of remission in Crohn's disease. Cochrane Database Syst. Rev. 10 (10), CD000545. 10.1002/14651858.CD000545.pub5 27783843 PMC6464152

[B15] Chen D. Chu B. Yang X. Liu Z. Jin Y. Kon N. (2021). iPLA2β-mediated lipid detoxification controls p53-driven ferroptosis independent of GPX4. Nat. Commun. 12 (1), 3644. 10.1038/s41467-021-23902-6 34131139 PMC8206155

[B16] Chen X. Li J. Kang R. Klionsky D. J. Tang D. (2021). Ferroptosis: Machinery and regulation. Autophagy 17 (9), 2054–2081. 10.1080/15548627.2020.1810918 32804006 PMC8496712

[B17] Chen X. Li M. Yan J. Liu T. Pan G. Yang H. (2017). Alcohol induces cellular senescence and impairs osteogenic potential in bone marrow-derived mesenchymal stem cells. Alcohol Alcohol 52 (3), 289–297. 10.1093/alcalc/agx006 28339869 PMC5397879

[B18] Choi W. M. Kim H. H. Kim M. H. Cinar R. Yi H. S. Eun H. S. (2019). Glutamate signaling in hepatic stellate cells drives alcoholic steatosis. Cell Metab. 30 (5), 877–889. 10.1016/j.cmet.2019.08.001 31474565 PMC6834910

[B19] Chu B. Kon N. Chen D. Li T. Liu T. Jiang L. (2019). ALOX12 is required for p53-mediated tumour suppression through a distinct ferroptosis pathway. Nat. Cell Biol. 21 (5), 579–591. 10.1038/s41556-019-0305-6 30962574 PMC6624840

[B20] Conrad M. Pratt D. A. (2019). The chemical basis of ferroptosis. Nat. Chem. Biol. 15 (12), 1137–1147. 10.1038/s41589-019-0408-1 31740834

[B21] Dahlmanns M. Yakubov E. Chen D. Sehm T. Rauh M. Savaskan N. (2017). Chemotherapeutic xCT inhibitors sorafenib and erastin unraveled with the synaptic optogenetic function analysis tool. Cell Death Discov. 3, 17030. 10.1038/cddiscovery.2017.30 28835855 PMC5541984

[B22] Dey A. Cederbaum A. I. (2006). Alcohol and oxidative liver injury. Hepatology 43 (2 1), S63–S74. 10.1002/hep.20957 16447273

[B23] Di Sario A. Candelaresi C. Omenetti A. Benedetti A. (2007). Vitamin E in chronic liver diseases and liver fibrosis. Vitam. Horm. 76, 551–573. 10.1016/S0083-6729(07)76021-1 17628189

[B24] Dixon S. J. Lemberg K. M. Lamprecht M. R. Skouta R. Zaitsev E. M. Gleason C. E. (2012). Ferroptosis: An iron-dependent form of nonapoptotic cell death. Cell 149 (5), 1060–1072. 10.1016/j.cell.2012.03.042 22632970 PMC3367386

[B25] Dixon S. J. Patel D. N. Welsch M. Skouta R. Lee E. D. Hayano M. (2014). Pharmacological inhibition of cystine-glutamate exchange induces endoplasmic reticulum stress and ferroptosis. Elife 3, e02523. 10.7554/eLife.02523 24844246 PMC4054777

[B26] Dixon S. J. Winter G. E. Musavi L. S. Lee E. D. Snijder B. Rebsamen M. (2015). Human haploid cell genetics reveals roles for lipid metabolism genes in nonapoptotic cell death. ACS Chem. Biol. 10 (7), 1604–1609. 10.1021/acschembio.5b00245 25965523 PMC4509420

[B27] Doll S. Freitas F. P. Shah R. Aldrovandi M. da Silva M. C. Ingold I. (2019). FSP1 is a glutathione-independent ferroptosis suppressor. Nature 575 (7784), 693–698. 10.1038/s41586-019-1707-0 31634899

[B28] Doll S. Proneth B. Tyurina Y. Y. Panzilius E. Kobayashi S. Ingold I. (2017). ACSL4 dictates ferroptosis sensitivity by shaping cellular lipid composition. Nat. Chem. Biol. 13 (1), 91–98. 10.1038/nchembio.2239 27842070 PMC5610546

[B29] Dolma S. Lessnick S. L. Hahn W. C. Stockwell B. R. (2003). Identification of genotype-selective antitumor agents using synthetic lethal chemical screening in engineered human tumor cells. Cancer Cell 3 (3), 285–296. 10.1016/s1535-6108(03)00050-3 12676586

[B30] Dong R. Wang X. Wang L. Wang C. Huang K. Fu T. (2021). Yangonin inhibits ethanol-induced hepatocyte senescence via miR-194/FXR axis. Eur. J. Pharmacol. 890, 173653. 10.1016/j.ejphar.2020.173653 33068587

[B31] El Hadi H. Vettor R. Rossato M. (2018). Vitamin E as a treatment for nonalcoholic fatty liver disease: Reality or myth? Antioxidants (Basel) 7 (1), 12. 10.3390/antiox7010012 29337849 PMC5789322

[B32] Fan B. Y. Pang Y. L. Li W. X. Zhao C. X. Zhang Y. Wang X. (2021). Liproxstatin-1 is an effective inhibitor of oligodendrocyte ferroptosis induced by inhibition of glutathione peroxidase 4. Neural Regen. Res. 16 (3), 561–566. 10.4103/1673-5374.293157 32985488 PMC7996026

[B33] Fang X. Cai Z. Wang H. Han D. Cheng Q. Zhang P. (2020). Loss of cardiac ferritin H facilitates cardiomyopathy via slc7a11-mediated ferroptosis. Circ. Res. 127 (4), 486–501. 10.1161/CIRCRESAHA.120.316509 32349646

[B34] Ferreira-Gonzalez S. Rodrigo-Torres D. Gadd V. L. Forbes S. J. (2021). Cellular senescence in liver disease and regeneration. Semin. Liver Dis. 41 (1), 50–66. 10.1055/s-0040-1722262 33764485

[B35] Friedmann Angeli J. P. Schneider M. Proneth B. Tyurina Y. Y. Tyurin V. A. Hammond V. J. (2014). Inactivation of the ferroptosis regulator Gpx4 triggers acute renal failure in mice. Nat. Cell Biol. 16 (12), 1180–1191. 10.1038/ncb3064 25402683 PMC4894846

[B36] Gao B. Xu M. J. Bertola A. Wang H. Zhou Z. Liangpunsakul S. (2017). Animal models of alcoholic liver disease: Pathogenesis and clinical relevance. Gene Expr. 17 (3), 173–186. 10.3727/105221617X695519 28411363 PMC5500917

[B37] Gautheron J. Gores G. J. Rodrigues C. M. P. (2020). Lytic cell death in metabolic liver disease. J. Hepatol. 73 (2), 394–408. 10.1016/j.jhep.2020.04.001 32298766 PMC7371520

[B38] Gu X. Liu Y. Dai X. Yang Y. G. Zhang X. (2023). Deciphering the potential roles of ferroptosis in regulating tumor immunity and tumor immunotherapy. Front. Immunol. 14, 1137107. 10.3389/fimmu.2023.1137107 36926345 PMC10011099

[B39] Harrison-Findik D. D. Klein E. Crist C. Evans J. Timchenko N. Gollan J. (2007). Iron-mediated regulation of liver hepcidin expression in rats and mice is abolished by alcohol. Hepatology 46 (6), 1979–1985. 10.1002/hep.21895 17763462

[B40] Harrison-Findik D. D. (2007). Role of alcohol in the regulation of iron metabolism. World J. Gastroenterol. 13 (37), 4925–4930. 10.3748/wjg.v13.i37.4925 17854133 PMC4434614

[B41] Huang C. Y. Chen L. J. Chen G. Chao T. I. Wang C. Y. (2022). SHP-1/STAT3-Signaling-Axis-Regulated coupling between BECN1 and SLC7A11 contributes to sorafenib-induced ferroptosis in hepatocellular carcinoma. Int. J. Mol. Sci. 23 (19), 11092. 10.3390/ijms231911092 36232407 PMC9570040

[B42] Ioannou G. N. Dominitz J. A. Weiss N. S. Heagerty P. J. Kowdley K. V. (2004). The effect of alcohol consumption on the prevalence of iron overload, iron deficiency, and iron deficiency anemia. Gastroenterology 126 (5), 1293–1301. 10.1053/j.gastro.2004.01.020 15131790

[B43] Ivancovsky-Wajcman D. Fliss-Isakov N. Salomone F. Webb M. Shibolet O. Kariv R. (2019). Dietary vitamin E and C intake is inversely associated with the severity of nonalcoholic fatty liver disease. Dig. Liver Dis. 51 (12), 1698–1705. 10.1016/j.dld.2019.06.005 31281067

[B44] Jia M. Zhang H. Qin Q. Hou Y. Zhang X. Chen D. (2021). Ferroptosis as a new therapeutic opportunity for nonviral liver disease. Eur. J. Pharmacol. 908, 174319. 10.1016/j.ejphar.2021.174319 34252441

[B45] Jiang H. Zhang X. Yang W. Li M. Wang G. Luo Q. (2022). Ferrostatin-1 ameliorates liver dysfunction via reducing iron in thioacetamide-induced acute liver injury in mice. Front. Pharmacol. 13, 869794. 10.3389/fphar.2022.869794 35496274 PMC9039014

[B46] Jiang X. Stockwell B. R. Conrad M. (2021). Ferroptosis: Mechanisms, biology and role in disease. Nat. Rev. Mol. Cell Biol. 22 (4), 266–282. 10.1038/s41580-020-00324-8 33495651 PMC8142022

[B47] Jiang Y. Zhang T. Kusumanchi P. Han S. Yang Z. Liangpunsakul S. (2020). Alcohol metabolizing enzymes, microsomal ethanol oxidizing system, cytochrome P450 2E1, catalase, and aldehyde dehydrogenase in alcohol-associated liver disease. Biomedicines 8 (3), 50. 10.3390/biomedicines8030050 32143280 PMC7148483

[B48] Jin H. Lian N. Bian M. Zhang C. Chen X. Shao J. (2018). Oroxylin A inhibits ethanol-induced hepatocyte senescence via YAP pathway. Cell Prolif. 51 (3), e12431. 10.1111/cpr.12431 29318697 PMC6528849

[B49] Kaur J. Shalini S. Bansal M. P. (2010). Influence of vitamin E on alcohol-induced changes in antioxidant defenses in mice liver. Toxicol. Mech. Methods 20 (2), 82–89. 10.3109/15376510903559950 20067348

[B50] Kim J. H. Lewin T. M. Coleman R. A. (2001). Expression and characterization of recombinant rat Acyl-CoA synthetases 1, 4, and 5. Selective inhibition by triacsin C and thiazolidinediones. J. Biol. Chem. 276 (27), 24667–24673. 10.1074/jbc.M010793200 11319222

[B51] Kong L. Z. Chandimali N. Han Y. H. Lee D. H. Kim J. S. Kim S. U. (2019). Pathogenesis, early diagnosis, and therapeutic management of alcoholic liver disease. Int. J. Mol. Sci. 20 (11), 2712. 10.3390/ijms20112712 31159489 PMC6600448

[B52] Koppula P. Zhuang L. Gan B. (2021). Cystine transporter slc7a11/xCT in cancer: Ferroptosis, nutrient dependency, and cancer therapy. Protein Cell 12 (8), 599–620. 10.1007/s13238-020-00789-5 33000412 PMC8310547

[B53] Kouroumalis E. Tsomidis I. Voumvouraki A. (2023). Iron as a therapeutic target in chronic liver disease. World J. Gastroenterol. 29 (4), 616–655. 10.3748/wjg.v29.i4.616 36742167 PMC9896614

[B54] Kwon M. Y. Park E. Lee S. J. Chung S. W. (2015). Heme oxygenase-1 accelerates erastin-induced ferroptotic cell death. Oncotarget 6 (27), 24393–24403. 10.18632/oncotarget.5162 26405158 PMC4695193

[B55] Lange M. Olzmann J. A. (2021). Ending on a sour note: Lipids orchestrate ferroptosis in cancer. Cell Metab. 33 (8), 1507–1509. 10.1016/j.cmet.2021.07.011 34348094

[B56] Lavine J. E. Schwimmer J. B. Van Natta M. L. Molleston J. P. Murray K. F. Rosenthal P. (2011). Effect of vitamin E or metformin for treatment of nonalcoholic fatty liver disease in children and adolescents: The TONIC randomized controlled trial. JAMA 305 (16), 1659–1668. 10.1001/jama.2011.520 21521847 PMC3110082

[B57] Lee H. Zandkarimi F. Zhang Y. Meena J. K. Kim J. Zhuang L. (2020). Energy-stress-mediated AMPK activation inhibits ferroptosis. Nat. Cell Biol. 22 (2), 225–234. 10.1038/s41556-020-0461-8 32029897 PMC7008777

[B58] Li L. X. Guo F. F. Liu H. Zeng T. (2022). Iron overload in alcoholic liver disease: Underlying mechanisms, detrimental effects, and potential therapeutic targets. Cell Mol. Life Sci. 79 (4), 201. 10.1007/s00018-022-04239-9 35325321 PMC11071846

[B59] Li S. He Y. Chen K. Sun J. Zhang L. He Y. (2021). RSL3 drives ferroptosis through NF-*κ*B pathway activation and GPX4 depletion in glioblastoma. Oxid. Med. Cell Longev. 2021, 2915019. 10.1155/2021/2915019 34987700 PMC8720588

[B60] Li Y. Deng Y. Tang Y. Yu H. Gao C. Liu L. (2014). Quercetin protects rat hepatocytes from oxidative damage induced by ethanol and iron by maintaining intercellular liable iron pool. Hum. Exp. Toxicol. 33 (5), 534–541. 10.1177/0960327113499168 23928830

[B61] Linhart K. Bartsch H. Seitz H. K. (2014). The role of reactive oxygen species (ROS) and cytochrome P-450 2E1 in the generation of carcinogenic etheno-DNA adducts. Redox Biol. 3, 56–62. 10.1016/j.redox.2014.08.009 25462066 PMC4297928

[B62] Liu C. Y. Wang M. Yu H. M. Han F. X. Wu Q. S. Cai X. J. (2020). Ferroptosis is involved in alcohol-induced cell death *in vivo* and *in vitro* . Biosci. Biotechnol. Biochem. 84 (8), 1621–1628. 10.1080/09168451.2020.1763155 32419644

[B63] Liu H. Forouhar F. Lin A. J. Wang Q. Polychronidou V. Soni R. K. (2022). Small-molecule allosteric inhibitors of GPX4. Cell Chem. Biol. 29 (12), 1680–1693.e9. 10.1016/j.chembiol.2022.11.003 36423641 PMC9772252

[B64] Liu J. Song X. Kuang F. Zhang Q. Xie Y. Kang R. (2021). NUPR1 is a critical repressor of ferroptosis. Nat. Commun. 12 (1), 647. 10.1038/s41467-021-20904-2 33510144 PMC7843652

[B65] Liu S. Y. Tsai I. T. Hsu Y. C. (2021). Alcohol-related liver disease: Basic mechanisms and clinical perspectives. Int. J. Mol. Sci. 22 (10), 5170. 10.3390/ijms22105170 34068269 PMC8153142

[B66] Llovet J. M. Ricci S. Mazzaferro V. Hilgard P. Gane E. Blanc J. F. (2008). Sorafenib in advanced hepatocellular carcinoma. N. Engl. J. Med. 359 (4), 378–390. 10.1056/NEJMoa0708857 18650514

[B67] Louvet A. Mathurin P. (2015). Alcoholic liver disease: Mechanisms of injury and targeted treatment. Nat. Rev. Gastroenterol. Hepatol. 12 (4), 231–242. 10.1038/nrgastro.2015.35 25782093

[B68] Lv Y. Wu M. Wang Z. Wang J. (2022). Ferroptosis: From regulation of lipid peroxidation to the treatment of diseases. Cell Biol. Toxicol. 10.1007/s10565-022-09778-2 36459356

[B69] Ma R. Chen J. Liang Y. Lin S. Zhu L. Liang X. (2017). Sorafenib: A potential therapeutic drug for hepatic fibrosis and its outcomes. Biomed. Pharmacother. 88, 459–468. 10.1016/j.biopha.2017.01.107 28122312

[B70] Magtanong L. Ko P. J. To M. Cao J. Y. Forcina G. C. Tarangelo A. (2019). Exogenous monounsaturated fatty acids promote a ferroptosis-resistant cell state. Cell Chem. Biol. 26 (3), 420–432. 10.1016/j.chembiol.2018.11.016 30686757 PMC6430697

[B71] Maiorino M. Conrad M. Ursini F. (2018). GPx4, lipid peroxidation, and cell death: Discoveries, rediscoveries, and open issues. Antioxid. Redox Signal 29 (1), 61–74. 10.1089/ars.2017.7115 28462584

[B72] Maras J. S. Das S. Sharma S. Sukriti S. Kumar J. Vyas A. K. (2018). Iron-overload triggers ADAM-17 mediated inflammation in severe alcoholic hepatitis. Sci. Rep. 8 (1), 10264. 10.1038/s41598-018-28483-x 29980709 PMC6035223

[B73] Masaldan S. Clatworthy S. A. S. Gamell C. Meggyesy P. M. Rigopoulos A. T. Haupt S. (2018). Iron accumulation in senescent cells is coupled with impaired ferritinophagy and inhibition of ferroptosis. Redox Biol. 14, 100–115. 10.1016/j.redox.2017.08.015 28888202 PMC5596264

[B74] Meroni M. Longo M. Rametta R. Dongiovanni P. (2018). Genetic and epigenetic modifiers of alcoholic liver disease. Int. J. Mol. Sci. 19 (12), 3857. 10.3390/ijms19123857 30513996 PMC6320903

[B75] Miotto G. Rossetto M. Di Paolo M. L. Orian L. Venerando R. Roveri A. (2020). Insight into the mechanism of ferroptosis inhibition by ferrostatin-1. Redox Biol. 28, 101328. 10.1016/j.redox.2019.101328 31574461 PMC6812032

[B76] Mohammed A. Abd Al Haleem E. N. El-Bakly W. M. El-Demerdash E. (2016). Deferoxamine alleviates liver fibrosis induced by CCl4 in rats. Clin. Exp. Pharmacol. Physiol. 43 (8), 760–768. 10.1111/1440-1681.12591 27168353

[B77] Mueller S. Peccerella T. Qin H. Glassen K. Waldherr R. Flechtenmacher C. (2018). Carcinogenic etheno DNA adducts in alcoholic liver disease: Correlation with cytochrome P-4502E1 and fibrosis. Alcohol Clin. Exp. Res. 42 (2), 252–259. 10.1111/acer.13546 29120493

[B78] Muller F. Lim J. K. M. Bebber C. M. Seidel E. Tishina S. Dahlhaus A. (2022). Elevated FSP1 protects KRAS-mutated cells from ferroptosis during tumor initiation. Cell Death Differ. 30, 442–456. 10.1038/s41418-022-01096-8 36443441 PMC9950476

[B79] Nagashimada M. Ota T. (2019). Role of vitamin E in nonalcoholic fatty liver disease. IUBMB Life 71 (4), 516–522. 10.1002/iub.1991 30592129

[B80] Nanji A. A. Yang E. K. Fogt F. Sadrzadeh S. M. Dannenberg A. J. (1996). Medium chain triglycerides and vitamin E reduce the severity of established experimental alcoholic liver disease. J. Pharmacol. Exp. Ther. 277 (3), 1694–1700. 8667240

[B81] Ohiro Y. Garkavtsev I. Kobayashi S. Sreekumar K. R. Nantz R. Higashikubo B. T. (2002). A novel p53-inducible apoptogenic gene, PRG3, encodes a homologue of the apoptosis-inducing factor (AIF). FEBS Lett. 524 (1-3), 163–171. 10.1016/s0014-5793(02)03049-1 12135761

[B82] Packer M. (2023). Potential interactions when prescribing SGLT2 inhibitors and intravenous iron in combination in heart failure. JACC Heart Fail 11 (1), 106–114. 10.1016/j.jchf.2022.10.004 36396554

[B83] Parker J. L. Deme J. C. Kolokouris D. Kuteyi G. Biggin P. C. Lea S. M. (2021). Molecular basis for redox control by the human cystine/glutamate antiporter system xc<sup/>. Nat. Commun. 12 (1), 7147. 10.1038/s41467-021-27414-1 34880232 PMC8654953

[B84] Parola M. Pinzani M. (2019). Liver fibrosis: Pathophysiology, pathogenetic targets and clinical issues. Mol. Asp. Med. 65, 37–55. 10.1016/j.mam.2018.09.002 30213667

[B85] Patel D. Kharkar P. S. Gandhi N. S. Kaur E. Dutt S. Nandave M. (2019). Novel analogs of sulfasalazine as system x(c)(-) antiporter inhibitors: Insights from the molecular modeling studies. Drug Dev. Res. 80 (6), 758–777. 10.1002/ddr.21557 31199023

[B86] Pinero F. Costa P. Boteon Y. L. Duque S. H. Marciano S. Anders M. (2018). A changing etiologic scenario in liver transplantation for hepatocellular carcinoma in a multicenter cohort study from Latin America. Clin. Res. Hepatol. Gastroenterol. 42 (5), 443–452. 10.1016/j.clinre.2018.03.014 29773419

[B87] Ramm G. A. Ruddell R. G. (2005). Hepatotoxicity of iron overload: Mechanisms of iron-induced hepatic fibrogenesis. Semin. Liver Dis. 25 (4), 433–449. 10.1055/s-2005-923315 16315137

[B88] Reinhardt C. Arena G. Nedara K. Edwards R. Brenner C. Tokatlidis K. (2020). AIF meets the CHCHD4/Mia40-dependent mitochondrial import pathway. Biochim. Biophys. Acta Mol. Basis Dis. 1866 (6), 165746. 10.1016/j.bbadis.2020.165746 32105825

[B89] Rouault T. A. (2003). Hepatic iron overload in alcoholic liver disease: Why does it occur and what is its role in pathogenesis? Alcohol 30 (2), 103–106. 10.1016/s0741-8329(03)00102-2 12957293

[B90] Schwarz K. Foltz C. M. (1999). Selenium as an integral part of factor 3 against dietary necrotic liver degeneration. Nutrition. 15(3):255. 10408880

[B91] Seiler A. Schneider M. Forster H. Roth S. Wirth E. K. Culmsee C. (2008). Glutathione peroxidase 4 senses and translates oxidative stress into 12/15-lipoxygenase dependent- and AIF-mediated cell death. Cell Metab. 8 (3), 237–248. 10.1016/j.cmet.2008.07.005 18762024

[B92] Seitz H. K. Bataller R. Cortez-Pinto H. Gao B. Gual A. Lackner C. (2018). Alcoholic liver disease. Nat. Rev. Dis. Prim. 4 (1), 16. 10.1038/s41572-018-0014-7 30115921

[B93] Shin D. Kim E. H. Lee J. Roh J. L. (2018). Nrf2 inhibition reverses resistance to GPX4 inhibitor-induced ferroptosis in head and neck cancer. Free Radic. Biol. Med. 129, 454–462. 10.1016/j.freeradbiomed.2018.10.426 30339884

[B94] Shintoku R. Takigawa Y. Yamada K. Kubota C. Yoshimoto Y. Takeuchi T. (2017). Lipoxygenase-mediated generation of lipid peroxides enhances ferroptosis induced by erastin and RSL3. Cancer Sci. 108 (11), 2187–2194. 10.1111/cas.13380 28837253 PMC5666033

[B95] Silva I. Rausch V. Seitz H. K. Mueller S. (2017). Does hypoxia cause carcinogenic iron accumulation in alcoholic liver disease (ALD)? Cancers (Basel) 9 (11), 145. 10.3390/cancers9110145 29068390 PMC5704163

[B96] Sripetchwandee J. Kongkaew A. Kumfu S. Chunchai T. Chattipakorn N. Chattipakorn S. C. (2023). Ferrostatin-1 and Z-VAD-FMK potentially attenuated Iron-mediated neurotoxicity and rescued cognitive function in Iron-overloaded rats. Life Sci. 313, 121269. 10.1016/j.lfs.2022.121269 36493877

[B97] Su Y. Zhao B. Zhou L. Zhang Z. Shen Y. Lv H. (2020). Ferroptosis, a novel pharmacological mechanism of anti-cancer drugs. Cancer Lett. 483, 127–136. 10.1016/j.canlet.2020.02.015 32067993

[B98] Sui X. Zhang R. Liu S. Duan T. Zhai L. Zhang M. (2018). RSL3 drives ferroptosis through GPX4 inactivation and ROS production in colorectal cancer. Front. Pharmacol. 9, 1371. 10.3389/fphar.2018.01371 30524291 PMC6262051

[B99] Susin S. A. Lorenzo H. K. Zamzami N. Marzo I. Snow B. E. Brothers G. M. (1999). Molecular characterization of mitochondrial apoptosis-inducing factor. Nature 397 (6718), 441–446. 10.1038/17135 9989411

[B100] Suzuki Y. Saito H. Suzuki M. Hosoki Y. Sakurai S. Fujimoto Y. (2002). Up-regulation of transferrin receptor expression in hepatocytes by habitual alcohol drinking is implicated in hepatic iron overload in alcoholic liver disease. Alcohol Clin. Exp. Res. 26 (8), 26S-31S–31S. 10.1097/01.ALC.0000026830.27338.23 12198371

[B101] Tan H. K. Yates E. Lilly K. Dhanda A. D. (2020). Oxidative stress in alcohol-related liver disease. World J. Hepatol. 12 (7), 332–349. 10.4254/wjh.v12.i7.332 32821333 PMC7407918

[B102] Teschke R. (2018). Alcoholic liver disease: Alcohol metabolism, cascade of molecular mechanisms, cellular targets, and clinical aspects. Biomedicines 6 (4), 106. 10.3390/biomedicines6040106 30424581 PMC6316574

[B103] Tong J. Lan X. T. Zhang Z. Liu Y. Sun D. Y. Wang X. J. (2023). Ferroptosis inhibitor liproxstatin-1 alleviates metabolic dysfunction-associated fatty liver disease in mice: Potential involvement of PANoptosis. Acta Pharmacol. Sin. 44 (5), 1014–1028. 10.1038/s41401-022-01010-5 36323829 PMC10104837

[B104] Tsai W. Y. Tsai R. Y. Liu C. C. Wu J. L. Wong C. S. (2016). Sulfasalazine attenuates ACL transection and medial menisectomy-induced cartilage destruction by inhibition of cystine/glutamate antiporter. J. Orthop. Res. 34 (4), 650–657. 10.1002/jor.23069 26466556

[B105] Tsukamoto H. (2002). Iron regulation of hepatic macrophage TNFalpha expression. Free Radic. Biol. Med. 32 (4), 309–313. 10.1016/s0891-5849(01)00772-9 11841920

[B106] Wang X. Chen Y. Wang X. Tian H. Wang Y. Jin J. (2021). Stem cell factor SOX2 confers ferroptosis resistance in lung cancer via upregulation of SLC7A11. Cancer Res. 81 (20), 5217–5229. 10.1158/0008-5472.CAN-21-0567 34385181 PMC8530936

[B107] Wu J. Xue R. Wu M. Yin X. Xie B. Meng Q. (2022). Nrf2-Mediated ferroptosis inhibition exerts a protective effect on acute-on-chronic liver failure. Oxid. Med. Cell Longev. 2022, 4505513. 10.1155/2022/4505513 35480867 PMC9036161

[B108] Wu M. Xu L. G. Li X. Zhai Z. Shu H. B. (2002). AMID, an apoptosis-inducing factor-homologous mitochondrion-associated protein, induces caspase-independent apoptosis. J. Biol. Chem. 277 (28), 25617–25623. 10.1074/jbc.M202285200 11980907

[B109] Xiong S. She H. Sung C. K. Tsukamoto H. (2003). Iron-dependent activation of NF-kappaB in kupffer cells: A priming mechanism for alcoholic liver disease. Alcohol 30 (2), 107–113. 10.1016/s0741-8329(03)00100-9 12957294

[B110] Xiong S. She H. Zhang A. S. Wang J. Mkrtchyan H. Dynnyk A. (2008). Hepatic macrophage iron aggravates experimental alcoholic steatohepatitis. Am. J. Physiol. Gastrointest. Liver Physiol. 295 (3), G512–G521. 10.1152/ajpgi.90327.2008 18599584 PMC2536779

[B111] Xu L. Liu Y. Chen X. Zhong H. Wang Y. (2023). Ferroptosis in life: To be or not to be. Biomed. Pharmacother. 159, 114241. 10.1016/j.biopha.2023.114241 36634587

[B112] Xue M. Tian Y. Sui Y. Zhao H. Gao H. Liang H. (2022). Protective effect of fucoidan against iron overload and ferroptosis-induced liver injury in rats exposed to alcohol. Biomed. Pharmacother. 153, 113402. 10.1016/j.biopha.2022.113402 36076527

[B113] Yagoda N. von Rechenberg M. Zaganjor E. Bauer A. J. Yang W. S. Fridman D. J. (2007). RAS-RAF-MEK-dependent oxidative cell death involving voltage-dependent anion channels. Nature 447 (7146), 864–868. 10.1038/nature05859 17568748 PMC3047570

[B114] Yamada N. Karasawa T. Wakiya T. Sadatomo A. Ito H. Kamata R. (2020). Iron overload as a risk factor for hepatic ischemia-reperfusion injury in liver transplantation: Potential role of ferroptosis. Am. J. Transpl. 20 (6), 1606–1618. 10.1111/ajt.15773 31909544

[B115] Yan H. F. Zou T. Tuo Q. Z. Xu S. Li H. Belaidi A. A. (2021). Ferroptosis: Mechanisms and links with diseases. Signal Transduct. Target Ther. 6 (1), 49. 10.1038/s41392-020-00428-9 33536413 PMC7858612

[B116] Yang W. S. Kim K. J. Gaschler M. M. Patel M. Shchepinov M. S. Stockwell B. R. (2016). Peroxidation of polyunsaturated fatty acids by lipoxygenases drives ferroptosis. Proc. Natl. Acad. Sci. U. S. A. 113 (34), E4966–E4975. 10.1073/pnas.1603244113 27506793 PMC5003261

[B117] Yang W. S. SriRamaratnam R. Welsch M. E. Shimada K. Skouta R. Viswanathan V. S. (2014). Regulation of ferroptotic cancer cell death by GPX4. Cell 156 (1-2), 317–331. 10.1016/j.cell.2013.12.010 24439385 PMC4076414

[B118] Yang Y. Zhu T. Wang X. Xiong F. Hu Z. Qiao X. (2022). ACSL3 and ACSL4, distinct roles in ferroptosis and cancers. Cancers (Basel). 14 (23), 5896. 10.3390/cancers14235896 36497375 PMC9739553

[B119] Yu Y. Jiang L. Wang H. Shen Z. Cheng Q. Zhang P. (2020). Hepatic transferrin plays a role in systemic iron homeostasis and liver ferroptosis. Blood 136 (6), 726–739. 10.1182/blood.2019002907 32374849 PMC7414596

[B120] Zeng F. Lan Y. Wang N. Huang X. Zhou Q. Wang Y. (2022). Ferroptosis: A new therapeutic target for bladder cancer. Front. Pharmacol. 13, 1043283. 10.3389/fphar.2022.1043283 36408230 PMC9669411

[B121] Zha L. Liu R. Sobue T. Kitamura T. Ishihara J. Kotemori A. (2021). Dietary acrylamide intake and the risk of hematological malignancies: The Japan public health center-based prospective study. Nutrients 13 (2), 590. 10.3390/nu13020590 33670108 PMC7916863

[B122] Zheng J. Conrad M. (2020). The metabolic underpinnings of ferroptosis. Cell Metab. 32 (6), 920–937. 10.1016/j.cmet.2020.10.011 33217331

[B123] Zhou Q. Li T. Qin Q. Huang X. Wang Y. (2022). Ferroptosis in lymphoma: Emerging mechanisms and a novel therapeutic approach. Front. Genet. 13, 1039951. 10.3389/fgene.2022.1039951 36406116 PMC9669386

[B124] Zhou Z. Ye T. J. Bonavita G. Daniels M. Kainrad N. Jogasuria A. (2019). Adipose-specific lipin-1 overexpression renders hepatic ferroptosis and exacerbates alcoholic steatohepatitis in mice. Hepatol. Commun. 3 (5), 656–669. 10.1002/hep4.1333 31061954 PMC6492478

[B125] Zhou Z. Ye T. J. DeCaro E. Buehler B. Stahl Z. Bonavita G. (2020). Intestinal SIRT1 deficiency protects mice from ethanol-induced liver injury by mitigating ferroptosis. Am. J. Pathol. 190 (1), 82–92. 10.1016/j.ajpath.2019.09.012 31610175 PMC6943377

[B126] Zhu L. Chen D. Zhu Y. Pan T. Xia D. Cai T. (2021). GPX4-Regulated ferroptosis mediates S100-induced experimental autoimmune hepatitis associated with the Nrf2/HO-1 signaling pathway. Oxid. Med. Cell Longev. 2021, 6551069. 10.1155/2021/6551069 34966478 PMC8712167

[B127] Zilka O. Shah R. Li B. Friedmann Angeli J. P. Griesser M. Conrad M. (2017). On the mechanism of cytoprotection by ferrostatin-1 and liproxstatin-1 and the role of lipid peroxidation in ferroptotic cell death. ACS Cent. Sci. 3 (3), 232–243. 10.1021/acscentsci.7b00028 28386601 PMC5364454

